# Enhanced agricultural carbon sinks provide benefits for farmers and the climate

**DOI:** 10.1038/s43016-024-01039-1

**Published:** 2024-09-23

**Authors:** Stefan Frank, Andrey Lessa Derci Augustynczik, Petr Havlík, Esther Boere, Tatiana Ermolieva, Oliver Fricko, Fulvio Di Fulvio, Mykola Gusti, Tamas Krisztin, Pekka Lauri, Amanda Palazzo, Michael Wögerer

**Affiliations:** 1 International Institute for Applied Systems Analysis, Laxenburg, Austria; 2 Department of Environmental Geography, Vrije Universiteit Amsterdam, Amsterdam, The Netherlands

**Keywords:** Climate-change mitigation, Environmental impact, Economics, Agriculture

## Abstract

Carbon sequestration on agricultural land, albeit long-time neglected, offers substantial mitigation potential. Here we project, using an economic land-use model, that these options offer cumulative mitigation potentials comparable to afforestation by 2050 at 160 USD_2022_ tCO_2_ equivalent (tCO_2_e^−1^), with most of it located in the Global South. Carbon sequestration on agricultural land could provide producers around the world with additional revenues of up to 375 billion USD_2022_ at 160 USD_2022_ tCO_2_e^−1^ and allow achievement of net-zero emissions in the agriculture, forestry and other land-use sectors by 2050 already at economic costs of around 80–120 USD_2022_ tCO_2_e^−1^. This would, in turn, decrease economy-wide mitigation costs and increase gross domestic product (+0.6%) by the mid-century in 1.5 °C no-overshoot climate stabilization scenarios compared with mitigation scenarios that do not consider these options. Unlocking these potentials requires the deployment of highly efficient institutions and monitoring systems over the next 5 years across the whole world, including sub-Saharan Africa, where the largest mitigation potential exists.

## Abstract (web summary)

Carbon sequestration on agricultural land holds great promise for combating climate change. This study estimates the mitigation potential of three sequestration practices—soil carbon enhancement, biochar application on cropland and silvo-pastoral systems—while identifying cost-effective mitigation portfolios.

## Main

The food system, including its value chains, is one of the key sources of greenhouse gases (GHG) and is estimated to account for one-third (16–18 GtCO_2_ equivalent (GtCO_2_e) per year) of global anthropogenic GHG emissions^1,2^ of which 11.9 ± 4.4 GtCO_2_e per year are attributed to agriculture, forestry and other land uses (AFOLU) over the period 2010–2019^3^. Given the importance of agriculture as a driver of tropical deforestation^4,5^ and its substantial share in current and projected emissions^6,7^, the speed and ambition of climate action in the sector is vital to stabilize the climate. Not only will it determine the level of residual GHG emissions and, hence, the requirement for negative emissions once carbon neutrality has been achieved^8^, but lack of mitigation action in the food system may preclude reaching the 1.5 °C target in the first place^9,10^.

Despite large cost-effective abatement potentials in agriculture^6,11–13^, there persists a reluctance of countries to adopt mandatory price-based mitigation policies in agriculture^10,14^. Even though many countries refer to agriculture in their nationally determined contributions, only New Zealand was planning to include agricultural emissions in its emission trading scheme as of 2025^10,15^ but recently changed its plans. Aside from challenges related to governance and high transaction, monitoring, reporting and verification costs^16–18^, concerns related to food security and increasing food prices if stringent agricultural mitigation efforts were adopted in vulnerable regions of the Global South^19–21^, and also, concerns related to poverty have been raised^22^.

Climate-smart agricultural practices have the potential to generate a substantial carbon sink^23–25^. Enhanced CO_2_ sequestration from soil conservation practices on agricultural land, such as improved fertilizer, tillage and residue management, or cover cropping (0.7–2.5 GtCO_2_e per year), biochar application (0.3–1.8 GtCO_2_e per year) and agroforestry (0.4–1.1 GtCO_2_e per year) are considered promising mitigation options and economically viable at GHG prices up to 100 USD_2015_ tCO_2_e^−1^ (ref. ^3^). Besides the direct benefit for the climate, these options could help to alleviate other socio-economic and environmental challenges as well^26^. Increasing the carbon content in soils, for example, via biochar application or soil conservation practices, can increase crop productivity under certain conditions, especially on degraded soils^27–32^. Agroforestry may increase resilience to climate change impacts and, hence, improve food security^33,34^ but can at the same time provide additional biomass for energy uses, thereby reducing harvest pressure from managed forests^35^. Hence, promoting the widespread adoption of climate-smart agricultural practices while considering equity principles can contribute to the wider Sustainable Development Goals^36^.

Still, CO_2_ sequestration options on agricultural land and their co-benefits have not been considered in the global mitigation pathways reviewed under the 6th Assessment Report of the Intergovernmental Panel on Climate Change, and their abatement potential was only assessed in isolation from other sectors and from the market dynamics^3^. While studies explored their isolated technical and economic mitigation potentials in bottom-up assessments^23–25,37^, economic implications for farmers and market rebound effects across options remain under researched^22,38,39^. Yet, these economic dynamics are important to avoid over- and/or underestimation of mitigation potentials/costs and are a prerequisite for the inclusion of these mitigation options in Integrated Assessment Models (IAMs).

Here, we apply an economic land-use model (Global Biosphere Management Model, GLOBIOM)^13,40^ enhanced with a new set of CO_2_ sequestration options on agricultural land (Methods) to assess economic implications and identify the cost-effective GHG mitigation option portfolios for agriculture under current climatic conditions. We evaluate the importance of CO_2_ sequestration on agricultural land within the broader AFOLU sector by linking with a forest sector model (Global Forest Model, G4M)^41,42^. We consider three novel agricultural CO_2_ sequestration practices that are widely discussed in literature^23–25,43^: (1) soil carbon (SOC) enhancement in cropland and pastures (for example, different tillage, fertilization or crop residue management practices, and so on), (2) the application of biochar on cropland and (3) the expansion of silvo-pastural systems. We do not consider agroforestry systems on cropland to preclude potential trade-offs with crop production^44,45^. Our baseline scenario (‘baseline’) is based on the Shared Socio-Economic Pathway 2^46,47^, which represents a middle-of-the road scenario with continuation of current trends (Supplementary Information). We then identify the cost-effective mitigation potentials considering market feedbacks and spillover effects across regions, sectors and mitigation options and quantify synergies and trade-offs of the assessed agricultural CO_2_ sequestration options with other economic and socio-economic outcomes. Our scenario assessment (Table 1) is built around two elements (Methods): (1) different GHG price trajectories that consider (‘agCO_2_’) or do not consider (‘default’) agricultural CO_2_ sequestration options under baseline bioenergy demands (these scenarios are used to estimate the cost-effective mitigation potentials) and (2) an alternative set of scenarios with enhanced biomass demands for bioenergy compatible with the 1.5 °C target (‘agCO_2__bio’ and ‘default_bio’). These scenarios are used to investigate the role of agricultural CO_2_ sequestration options for achieving net zero AFOLU emissions in the context of the 1.5 °C target.

**Table 1:** Quantified scenario matrix in GLOBIOM–G4M and its specifications

Scenario acronym	AGRI N_2_O and CH_4_	AGRI CO_2_	FOLU CO_2_	Bioenergy demand
baseline	✗	✗	✗	Baseline
default	✓	✗	✓	Baseline
agCO_2_	✓	✓	✓	Baseline
default_bio	✓	✗	✓	1.5 °C
agCO_2__bio	✓	✓	✓	1.5 °C

GHG price trajectories are implemented on different GHG sources: AGRI N_2_O and CH_4_, nitrous oxide emissions from the application of organic and synthetic fertilizers and manure management, methane emissions from manure management, enteric fermentation and rice cultivation; AGRI CO_2_, carbon dioxide emissions/removals on agricultural land; FOLU CO_2_, carbon dioxide emissions/removals from land-use change and forestry.

Finally, to assess the robustness of our results, we conduct a sensitivity analysis where we test alternative parameterizations for the agricultural CO_2_ sequestration options in the model regarding the costs of adoption, the maximum adoption levels, the time it takes to reach the new carbon stock equilibrium following the adoption of a practice, the number of trees in silvo-pastures and the future demand for livestock products. The aim of this study is to bring new insights on the economic opportunities of agricultural CO_2_ sequestration options and related socio-economic trade-offs/synergies, and to provide a dataset for the integration of these options into IAMs.

## Results

### Carbon sequestration potentials on agricultural land

To explore the economic mitigation potential of three agricultural CO_2_ sequestration options within the overall land-based GHG mitigation potential, we contrast results of the ‘agCO_2_’ scenario with a GHG price on all AFOLU emissions/removals to a ‘baseline’ scenario without land-based mitigation efforts. Applying a linearly increasing GHG price that reaches 160 (80 and 240) USD_2022_ tCO_2_e^−1^ by 2050, we find that these options can provide a substantial carbon sink on agricultural land of up to 2.8 (1.6 and 2.5) GtCO_2_e per year by 2050. The smaller GHG mitigation potential at 240 USD_2022_ tCO_2_e^−1^ is explained by the enhanced uptake of CO_2_ sequestration practices and mitigation early on when moving towards higher GHG prices. At 160 USD_2022_ tCO_2_e^−1^ by 2050, the estimated CO_2_ sequestration potential on agricultural land represents already 36–41% of the expected total AFOLU GHG mitigation requirements including forests of around 7–8 GtCO_2_e per year in existing IAM based 1.5 °C climate stabilization scenarios^8,48,49^.

**Fig. 1:**
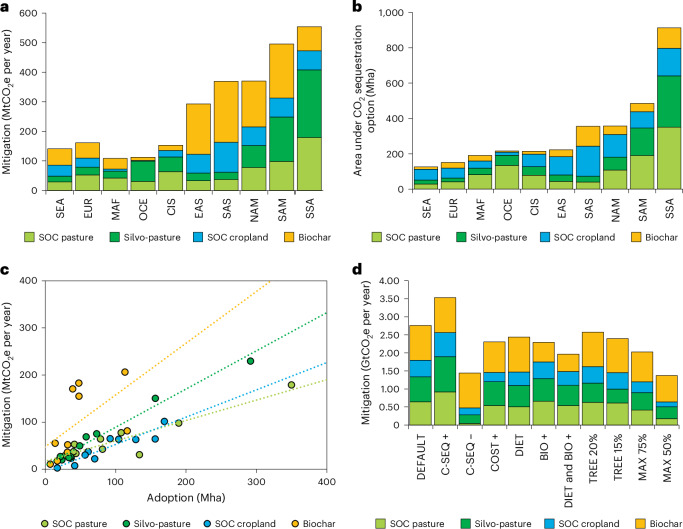
Adoption of agricultural CO_2_ sequestration options in the ‘agCO_2_’ scenario with a GHG price of 160 USD_2022_ tCO_2_e^−1^ by 2050. **a**, The GHG mitigation potential of different CO_2_ sequestration options. **b**, The area under the different options required to meet these mitigation potentials across world regions. **c**, Mitigation efficiency across options. **d**, Mitigation potential across sensitivity scenarios. ‘C-SEQ +’, more sustained (additional 10 years) CO_2_ sequestration for SOC sequestration options and silvo-pastures; ‘C-SEQ −’, more limited (10 years less) CO_2_ sequestration for SOC sequestration options and silvo-pastures; ‘COST +’, doubling of adoption costs for all agricultural CO_2_ sequestration options; ‘DIET’, reduced livestock consumption in Western countries; ‘BIO +’, increased bioenergy demand compatible with 1.5 **°**C target; ‘TREE 20%’, 20% of silvo-pasture system covered with trees instead of 25%; ‘TREE 15%’, 15% of silvo-pasture system covered with trees instead of 25%; ‘MAX 75%’, limit maximum adoption potential of agricultural CO_2_ sequestration options to 75% of default; ‘MAX 50%’, limit maximum adoption potential of agricultural CO_2_ sequestration options to 50% of default; NAM, North America; SAM, South and Central America; CIS, former Soviet Union; EUR, Europe; EAS, East Asia; SAS, South Asia; SEA, Southeast Asia; OCE, Oceania; MAF, Middle East and Northern Africa; SSA, Southern Africa.

Globally, 1.1 GtCO_2_e per year (39%) of the CO_2_ sequestration potential on agricultural land is sourced from the adoption of practices on cropland and grasslands to enhance SOC, 1.0 GtCO_2_e per year (35%) from the application of biochar to cropland and 0.7 GtCO_2_e per year (26%) from transforming pastures to silvo-pastures by planting trees (Fig. 1d). Over time, we first observe the uptake of improved crop- and grassland management practices for enhanced SOC sequestration due to low adoption costs and its benefits for agricultural productivities that amplify the cost-efficiency of these measures, as well as the transformation of pastures to silvo-pastoral systems. Biochar, however, only becomes economically viable at higher GHG prices related to feedstock prices and the competition for biomass as input for the pyrolysis with other energy and non-energy uses.

Across world regions, sub-Saharan Africa is projected to have the largest cost-effective CO_2_ sequestration potential on agricultural land at a GHG price of 160 USD_2022_ tCO_2_e^−1^, followed by Latin America (Fig. 1a). While CO_2_ sequestration options targeting grasslands are primarily located in sub-Saharan Africa (37% of global silvo-pasture area and 32% of improved pasture SOC sequestration practices) and Latin America (20% of global silvo-pasture area and 17% of improved pasture SOC sequestration practices), improved cropland management practices are also largely adopted in South Asia (19% of global area) and North America (14% of global area). Overall, 27% of the cost-effective CO_2_ sequestration potential at 160 USD_2022_ tCO_2_e^−1^ is located in the Global North, compared with 73% in the Global South.

To realize the estimated mitigation potentials of 2.8 GtCO_2_e per year by 2050, globally 780 Mha of silvo-pastures are being established (43% of managed grassland), conservation agriculture is adopted on 900 Mha (53% of total cropland) and improved grassland SOC management practices on 1,100 Mha (60% of managed grassland) (Fig. 1b). In terms of sequestration per hectare (see Supplementary Table 3 for regional details), biochar delivers the highest return at global scale (Fig. 1c) generating on average carbon sinks of around 2.1 tCO_2_e ha^−1^, followed by silvo-pastures (0.9 tCO_2_e ha^−1^) and SOC sequestration practices (0.5–0.6 tCO_2_e ha^−1^).

To put the estimated adoption potentials into context with existing literature, Zomer et al.^50^ calculated that around half of the global agricultural area has fairly low biomass carbon stocks below 10 tC ha^−1^ that could be elevated substantially by (already incremental) increases in tree coverage^51^, and Prestele et al.^52^ estimate a technical adoption potential of conservation agriculture somewhere between 38% and 81% of arable land. Several studies also anticipate large potential of improved pasture management and restoration practices^53–55^ given that half of the global grassland area has been degraded to some extent^56^. Biochar is applied on some 460 Mha of cropland (28%) requiring 3,300 Mm^3^ biomass as input for the pyrolysis, which is mainly sourced from crop residues (54%) and silvo-pastures (34%) and, to a smaller extent, from forestry residues (10%) and short rotation tree plantations (2%). To put this into context, total biomass demand for bioenergy is estimated at 8,300 Mm^3^ in the baseline scenario and 15,900 Mm^3^ in a 1.5 °C compatible scenario by 2050.

The estimated cost-effective GHG mitigation potentials are interlinked with socio-economic and bio-physical scenario drivers and assumptions that affect the cost-efficiency of these options. For example, limiting the adoption potentials across options or varying the sequestration rates across mitigation options is found to have profound impact on the GHG mitigation potentials from agricultural CO_2_ sequestration options (from −27% to −50%, Fig. 1d). A scenario with dietary changes towards less livestock-based products in Western countries suggests more limited mitigation potentials within the agricultural sector for grassland SOC (−21%), cropland SOC (−18%) and silvo-pastures (−15%) due to the overall decline in agricultural production and abandonment of agricultural areas in response to the diet shift (though delivering additional GHG mitigation of non-CO_2_ gases and enhanced FOLU CO_2_ sequestration). If such a diet scenario is combined with 1.5 °C compatible bioenergy demands, total CO_2_ sequestration on agricultural land is even more reduced (−29%). To achieve climate neutrality, a diverse portfolio of mitigation options will need to be deployed with multiple inter-dependencies. Our results highlight the inter-dependencies across mitigation options and the importance of integrated assessments to avoid overestimation of the cost-effectiveness or GHG mitigation potentials of individual options.

### Net zero AFOLU emissions

**Fig. 2:**
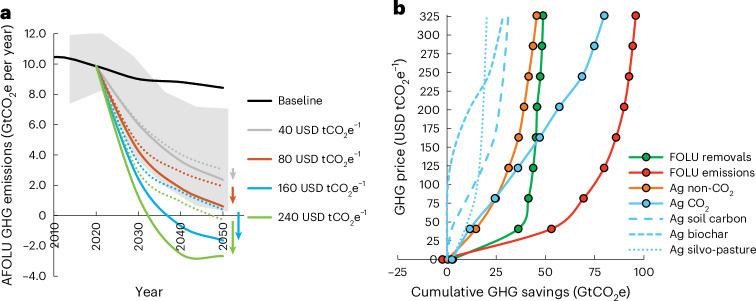
AFOLU GHG emissions and cumulative GHG mitigation across different GHG price scenarios up to 2050. **a**, AFOLU GHG emissions across GHG price scenarios over time with (the solid lines represent ‘agCO_2__bio’) and without consideration of CO_2_ sequestration practices on agricultural land (the dotted lines represent ‘default_bio’). The values below zero indicate net negative AFOLU emission levels. The grey area indicates minimum and maximum AFOLU emission ranges across IAMs for a peak warming 1.5 °C scenario from Hasegawa et al.^48^, and the arrows indicate additional GHG mitigation when considering agricultural CO_2_ sequestration options across GHG price scenarios. **b**, Cumulative AFOLU GHG mitigation from 2020–2050 for different GHG sources at different GHG prices up to 325 USD_2022_ tCO_2_e^−1^ by 2050 (‘agCO_2__bio’).

To assess the potential of CO_2_ sequestration practices to achieve net zero AFOLU emissions and thereby contributing to 1.5 °C climate stabilization efforts, we contrast the ‘agCO_2__bio’ scenario (that reaches a 1.5 °C compatible AFOLU emission trajectory and considers enhanced biomass demands for bioenergy) to the ‘default_bio’ that does not consider agricultural CO_2_ sequestration options. Overall, CO_2_ sequestration options allow us to achieve deeper emission reductions over the next decades and consequently net negative AFOLU emissions at lower GHG prices (Fig. 2a). Applying a GHG price of 160 USD_2022_ tCO_2_e^−1^ allows to achieve negative AFOLU emissions (−1.6 GtCO_2_e per year) by 2050 when considering these options as compared with 0.4 GtCO_2_e per year without. More than half of the mitigation in 2050 is sourced from the FOLU sector (5.9 GtCO_2_e per year, 57%), followed by CO_2_ sequestration on agricultural land (2.3 GtCO_2_e per year, 23%) and the reduction of agricultural non-CO_2_ gases (2.1 GtCO_2_e per year, 20%). Even at lower GHG prices of 80–120 USD_2022_ tCO_2_e^−1^, these options would enable to reduce AFOLU emissions to around from 0.6 to −0.9 GtCO_2_e per year by 2050, which would be compatible, and even below, existing 1.5 °C climate stabilization scenarios that require on average AFOLU GHG emissions to drop to around 3 GtCO_2_e per year by 2050 (refs. ^8,48^).

Especially for 1.5 °C climate stabilization scenarios with peak warming by 2050 (ref. ^49^), agricultural CO_2_ sequestration options can make an important difference in the economy-wide mitigation option portfolio and reduce mitigation costs in MESSAGEix-GLOBIOM^57^. Considering these novel CO_2_ options in the model shows that GHG prices could drop by 48% by 2050 with positive effects on global gross domestic product (GDP) (+0.6% by 2050) as compared with a 1.5 °C scenario without agricultural CO_2_ sequestration options as counterfactual. These effects are less pronounced when moving towards the end of the century or in less ambitious 2 °C peak warming scenarios. Therefore, particularly in ambitious stabilization scenarios that approach the asymptote of the economy-wide marginal abatement cost curve where any additional mitigation comes with substantial (economic) costs, agricultural CO_2_ sequestration options can make an important contribution to reduce those costs.

Looking at cumulative AFOLU GHG mitigation from 2020 to 2050 (Fig. 2b), agricultural CO_2_ sequestration options can provide similar mitigation potentials at the global scale at GHG prices >160 USD_2022_ tCO_2_e^−1^ as compared with other important AFOLU options modelled in this study, such as enhanced FOLU carbon sequestration via afforestation and reforestation or the reduction of agricultural non-CO_2_ emissions. At a GHG price of 160 USD_2022_ tCO_2_e^−1^, model results indicate that SOC sequestration practices on cropland and grasslands could provide cumulative GHG savings of 24 GtCO_2_e up to 2050, the establishment of silvo-pastures 18 GtCO_2_e, while the application of biochar to cropland soils contributes only 5 GtCO_2_e by 2050. Since the economic mitigation potential from biochar application is conditional on the biomass demands in other sectors that compete for the biomass feedstock, considering 1.5 °C compatible biomass demands for bioenergy production almost halves the economic mitigation potential of biochar (Fig. 1d) due to increased competition for the biomass resource and higher biomass prices. In addition, while other GHG mitigation sources such as FOLU removals tend to saturate at GHG prices >160 USD_2022_ tCO_2_e^−1^, the carbon sink from agricultural CO_2_ sequestration practices continues to increase providing 65% higher mitigation compared with the FOLU sink at 325 USD_2022_ tCO_2_e^−1^.

**Fig. 3:**
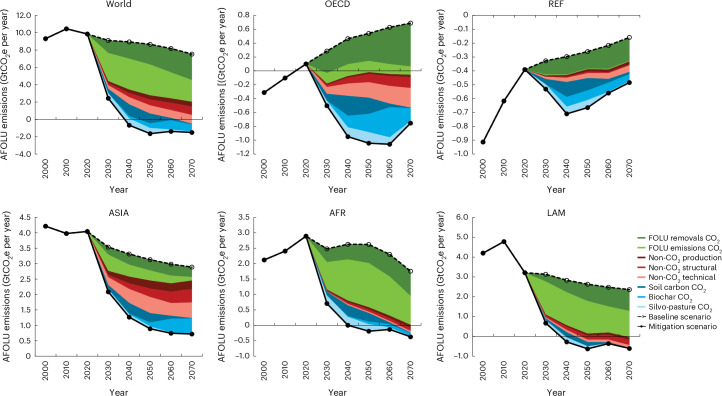
Global and regional AFOLU mitigation potentials in the ‘agCO_2__bio’ scenario applying a GHG price of 160 (270) USD_2022_ tCO_2_e^−1^ by 2050 (2070) on AFOLU GHG emissions/removals compared with the ‘baseline’ scenario. FOLU removals CO_2_, removals from afforestation and reforestation, forest management and other land-use changes; FOLU emissions CO_2_, emissions from deforestation, forest management and other land-use changes; non-CO_2_ production, CH_4_ and N_2_O emission change from changes in agricultural production levels; non-CO_2_ structural, CH_4_ and N_2_O emission change from structural changes in agriculture such as international trade; non-CO_2_ technical, CH_4_ and N_2_O emission change from adoption of technical mitigation options; soil carbon CO_2_, CO_2_ sequestration in soils from improved cropland and grassland management; biochar CO_2_, CO_2_ sequestration from biochar application; silvo-pasture CO_2_, CO_2_ sequestration from silvo-pastures; OECD, OECD countries; REF, former Soviet Union; ASIA, Asia; AFR, Africa; LAM, Latin America.

Though absolute mitigation potentials of agricultural CO_2_ sequestration options are mostly located in the Global South, the relative importance within the overall cost-effective AFOLU mitigation option portfolio varies across world regions (Fig. 3). In the countries of the Global South, those options contribute on average some 18% of the AFOLU GHG mitigation potential at 160 USD_2022_ tCO_2_e^−1^ by 2050 (11% in Latin America and 21% in Africa) given the large cost-effective mitigation potentials from reducing land-use change emissions including emissions from deforestation. However, in the Global North, these practices represent with 44% a much larger share of the total AFOLU abatement at 160 USD_2022_ tCO_2_e^−1^.

In addition, CO_2_ sequestration options retain more agricultural land under production under stringent mitigation efforts as they pay farmers for the carbon sink they provide. Enhanced CO_2_ sequestration on grassland and the establishment of silvo-pastures improves the GHG efficiency of rather GHG intensive pasture-based livestock production systems in tropical countries^58^. Consequently, more pasture-based livestock production systems remain competitive, even under a GHG price and the abandonment of pastures and subsequent reforestation slightly declines (−210 Mha, 85% of which is in Latin America and sub-Saharan Africa). Hence, the inclusion of agricultural CO_2_ sequestration options in AFOLU mitigation efforts may relieve some of the socio-economic challenges in the Global South related to agricultural land abandonment in response to a GHG price.

### Economic implications for farmers

**Fig. 4:**
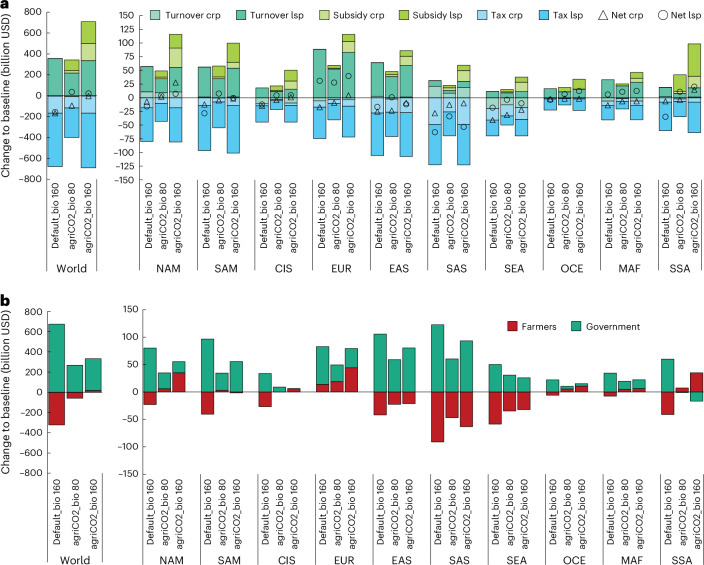
Economic impact on farmers across different GHG mitigation scenarios. **a**, The change in agricultural turnover and GHG price (subsidy and tax) effects across scenarios in 2050 compared with the baseline scenario. Gross turnover for crop (Turnover crp) and livestock (Turnover lsp) products is calculated by multiplying changes in agricultural prices and production quantities compared with the baseline. GHG tax (Tax crp and Tax lsp) and subsidy (Subsidy crp, Subsidy lsp) effects are calculated multiplying emissions or removals from agriculture with the GHG price of 80 or 160 USD_2022_ tCO_2_e^−1^. Net turnover (Net crp and Net lsp) for farmers is calculated as the sum of turnover, tax and subsidy effects. **b**, Net turnover effects for farmers (+ turnover − tax + subsidy effects) and government budgets (+ tax − subsidy effects) across scenarios in 2050. default_bio 160 – 160 USD_2022_ tCO_2_e^−1^ GHG price on all AFOLU emissions/removals except CO_2_ on agricultural land, agCO_2__bio 80 and agCO_2__bio 160 scenarios: 80 or 160 USD_2022_ tCO_2_e^−1^ GHG price on all AFOLU emissions including CO_2_ on agricultural land. NAM, North America; SAM, South and Central America; CIS, former Soviet Union; EUR, Europe; EAS, East Asia; SAS, South Asia; SEA, Southeast Asia; OCE, Oceania; MAF, Middle East and Northern Africa; SSA, Southern Africa.

Agricultural CO_2_ sequestration practices could also provide an interesting source of revenues in the future to farmers if they were paid for the carbon sink they generate. GHG prices are currently not considered the policy instrument of choice for agriculture. However, if a GHG tax were applied also in agriculture following the polluter pays principle, agricultural CO_2_ sequestration options would enable producers to offset some of the economic losses from the GHG tax on non-CO_2_ emissions. Figure 4 displays economic impacts on producers under a GHG pricing scheme for three alternative mitigation scenarios with and without consideration of CO_2_ sequestration options on agricultural land compared with the ‘baseline’ scenario without mitigation policy. The three scenarios include the default mitigation scenario based on (1) existing AFOLU abatement options in IAMs (‘default_bio’, with AFOLU emissions of 0.4 GtCO_2_e per year by 2050 at 160 USD_2022_ tCO_2_e^−1^), (2) a scenario applying the same GHG prices but considering agricultural CO_2_ sequestration options, thus reaching higher GHG mitigation (‘agCO_2__bio 160’, −1.6 GtCO_2_e per year by 2050 at 160 USD_2022_ tCO_2_e^−1^) and (3) a scenario considering agricultural CO_2_ sequestration options and delivering similar GHG mitigation by 2050 as the ‘default_bio’ scenario (‘agCO_2__bio 80’, 0.6 GtCO_2_e per year by 2050 at 80 USD_2022_ tCO_2_e^−1^).

Results show that, on a global scale, producers are less impacted if CO_2_ sequestration options on agricultural land are subsidized under the mitigation policy (‘agCO_2__bio 80’ and ‘agCO_2__bio 160’). Agricultural gross turnover is higher in the scenario without CO_2_ sequestration on agricultural land (350 billion USD_2022_ in the ‘default_bio’ scenario compared with the baseline) as compared with the scenarios including CO_2_ sequestration options (210 billion USD_2022_ ‘agCO_2__bio 80’ and 330 billion USD_2022_ ‘agCO_2__bio 160’) as food price increases are less pronounced in the latter scenarios and more land remains used for agricultural purposes due to yield co-benefits of carbon sequestration options. GHG tax payments on agricultural emissions amount to some 675 billion USD_2022_ at 160 USD_2022_ tCO_2_e^−1^, and hence, producers experience net economic turnover losses of around 325 billion USD_2022_ in the ‘default_bio’ scenario (Fig. 4a).

In the scenarios with carbon sequestration incentives, producers can largely compensate the non-CO_2_ related GHG tax payments (390 billion USD_2022_ ‘agCO_2__bio 80’ and 690 billion USD_2022_ ‘agCO_2__bio 160’) with carbon credits from CO_2_ sequestration on agricultural land, which generates, if compensated with the GHG price additional revenues, around 125/375 billion USD_2022_ at 80/160 USD_2022_ tCO_2_e^−1^. Hence, in the ‘agCO_2__bio 80’ scenario, net economic turnover losses can be reduced to 55 billion USD_2022_ as compared with the baseline scenario in 2050, while in ‘agCO_2__bio 160’, slightly positive effects of around 15 billion USD_2022_ are noted. Net turnover gains are most pronounced in ‘agCO_2__bio 160’ for livestock producers in Oceania (+43% change compared with the baseline scenario in 2050), Europe (+13%) and sub-Saharan Africa (+9%) and for crop producers in North America (+14%). Similarly, positive economic effects for producers, though less pronounced, are observed for the ‘agCO_2__bio 80’ scenario.

Overall, we estimate global net revenues for producers of 70/235 billion USD_2022_ (at 80/160 USD_2022_ tCO_2_e^−1^) from the adoption of agricultural CO_2_ sequestration options by deducting the economic costs (area under the marginal abatement cost curve) of CO_2_ sequestration practices (55/140 billion USD_2022_) from the additional revenues generated from the GHG price (125/375 billion USD_2022_). Figure 4b presents the economic implications for farmers and governmental budgets. Considering agricultural CO_2_ sequestration options in the mitigation policy impacts government budgets as part of the revenues generated by the GHG tax are assumed to be transferred back to farmers by paying them for the generated carbon sinks on agricultural land. This, in turn, reduces the overall space for distributional measures of governments to, for example, consumers, as the net GHG price revenues (tax minus subsidy payments) would decline from 675 billion USD_2022_ (‘default_bio’) to only 265 (‘agCO_2__bio 80’) and 315 billion USD_2022_ (‘agCO_2__bio 160’) at global scale. Across regions, for sub-Saharan Africa carbon, subsidies exceed GHG tax revenues and, hence, have negative implications for the government budget (−17 billion USD_2022_) at a GHG price of 160 USD_2022_ tCO_2_e^−1^. These results warrant further examination at a less aggregate scale and highlight the importance of climate finance mechanism.

## Discussion and conclusions

To keep the 1.5 **°**C target within reach, the land-use sector including agriculture will have to contribute substantially to mitigation efforts^9,14,48^. We find that enhanced CO_2_ sequestration practices on agricultural land may generate a global carbon sink of up to 2.8 GtCO_2_e per year by 2050 at 160 USD_2022_ tCO_2_e^−1^, with the majority located in the Global South. Our estimates are smaller compared with bottom-up studies^25,51,59^ owing to the consideration of interlinkages across options and economic dynamics. However, the estimated potentials still represent 36–41% of the expected GHG mitigation requirements (7–8 GtCO_2_e per year by 2050) for the AFOLU sector in existing 1.5 °C climate stabilization scenarios^8,48^. Consequently, AFOLU emissions could be reduced to −1.6 GtCO_2_e per year by 2050 at 160 USD_2022_ tCO_2_e^−1^ if agricultural CO_2_ sequestration options were deployed jointly with other land-based mitigation options. The estimated CO_2_ sequestration potentials on agricultural land are subject to uncertainty and interlinked with other drivers such as biomass demand for bioenergy. Varying assumptions related to the maximum adoption potential and saturation time had profound effects on mitigation potentials. Considering 1.5 °C compatible bioenergy demands reduced the overall cost-effective mitigation potential of agricultural carbon sequestration by 17% to 2.3 GtCO_2_e per year at 160 USD_2022_ tCO_2_e^−1^ and almost halves the economic mitigation potential of biochar given increased competition for biomass. Climate stabilization pathways that highlight the importance of bioenergy for fossil fuel substitution and carbon capture and storage^60^ need to consider these potential trade-offs. Our results highlight the benefits of integrated economic assessments to capture trade-offs systematically and avoid overestimating the effectiveness of individual mitigation options.

CO_2_ sequestration options on agricultural land allow us to achieve deeper emission savings in the land-use sector over the coming decades and deliver economic benefits by reducing the economy-wide costs of climate mitigation. When moving towards GHG prices >160 USD_2022_ tCO_2_e^−1^, these options offer similar GHG mitigation potentials as other important AFOLU mitigation sources, such as increased afforestation and reforestation or the reduction of agricultural non-CO_2_ emissions. Consequently, the land-use sector could achieve net zero AFOLU emissions already at GHG prices around 80–120 USD_2022_ tCO_2_e^−1^ by 2050 (without considering transaction costs) if all AFOLU mitigation options were deployed jointly. However, agricultural CO_2_ sequestration options can accumulate carbon only over a limited period of time^59^. Across economic sectors, considering these options in the mitigation portfolio reduces GHG prices by 48% and increases global GDP by 0.6% in 2050 in a 1.5 °C scenario without temperature overshoot. CO_2_ sequestration options on agricultural land may also provide an essential source of income for farmers if remunerated accordingly. At 160 USD_2022_ tCO_2_e^−1^ farmers could receive carbon subsidies of 375 billion USD_2022_, which exceeds current direct transfers to farmers amounting to USD_2022_ 293 billion per year from 2019 to 2021^61^. Still, economic impacts vary across regions, and some of the regions providing the biggest mitigation potential in the Global South would also have to bear the largest costs. These findings highlight the importance of considering equity aspects and climate justice across world regions^62–66^.

The presented results should be considered within model and parameter uncertainties. For example, our scenario analysis is preformed given current climatic conditions and does not consider climate impacts and disturbances. Though exact magnitudes remain uncertain^67^, climate impacts can directly decrease the capacity of soils to store carbon by modifying plant carbon inputs and microbial processes, thereby affecting carbon stocks^55,68^ or indirectly affecting the carbon cycle via extreme weather events, such as floods or fires^69,70^. In addition, we assumed optimal fertilization rates for silvo-pastures, while fertilization rates may deviate from these optimal levels in practice. Especially in regions with soil nitrogen deficit, plantations in tree mixtures with nitrogen-fixing species may help to mitigate nutrient imbalances^71,72^. These aspects deserve further investigation in future studies. Besides, our economic cost estimates are on the optimistic side, as certain costs, such as transaction costs, institutional costs and implementation costs, are not accounted for in our modelling framework, which would decrease cost-effectiveness of these options, especially for regions where sequestration rates are low^17^. Though implementation cost estimates vary widely in literature, these can be as high as 65–85% of the total carbon credit cost for an agricultural offset scheme in Western Canada^73^. Several structural, institutional or social and behavioural barriers need to be overcome before realizing the estimated mitigation potentials^17,74^, such as uncertainty on short-term adoption potentials given farm structure, land tenure rights or inertia of land owners, high monitoring, reporting and verification costs that impede adoption beyond large companies and farms or lack of institutional capacity to enforce policy targets^74–77^. Together with risks related to performance and additionality of generated carbon sinks^17^, this makes large-scale uptake of these options and inclusion in a policy scheme at a global scale rather unlikely in the short term.

Agricultural mitigation policies should be designed in an integrated and coordinated manner across gases, sectors and world regions to avoid rebound or leakage effects^38,75,78^. However, since the time it takes to prepare, formulate and adopt agricultural policies is probably the number of years left to keep the 1.5 °C target feasible with current emission levels^79^ (for example, a first legislative proposal for the EU Common Agriculture Policy for the period 2023–2027 was published by the European Commission in 2018^80^), agricultural mitigation policy design would need to see unprecedented fast tracking to bring any substantial benefits already by 2030.

Given the large variation in emission intensity across agricultural commodities and countries^58,73,81,82^, mitigation policies should prioritize commodities with high emission intensities. The ruminant sector is an interesting lever from a policy perspective as production is GHG intensive, but it offers large cost-effective GHG mitigation potentials^6,20,83,84^, as also assessed in this study. Developing best-practice policies by 2030 targeting these GHG intensive commodities in countries with strong institutional capacity with the possibility to up- and out-scale once operational to other countries should be among the priorities. Still, the structure of the livestock sector with a large number of smallholders^58,85^ complicates the implementation of monitoring, reporting and verification systems^73^, which is crucial to ensure effectiveness of the mitigation policy and to identify and correct potential negative policy effects timely. Here, targeting key players within the supply chain could facilitate policy implementation and deliver sizeable emission reductions^73^. For example, the 60 largest companies listed in the Coller FAIRR Protein Producer Index cover approximately 20% of the global livestock and aquaculture market with high dominance in some regional markets, that is, China, where they represent nearly 30% of the Chinese market for animal proteins and 100% of the domestic dairy market (www.fairr.org/resources/reports/coller-fairr-protein-producer-index-2018), and reaching those alone could, thus, bring substantial benefits with limited implementation costs. In addition, unlike smallholders, these companies are more probable to have the capacity to deal with the monitoring, reporting and verification and bear those costs.

Finally, the creation of carbon sinks should be remunerated and included in a policy scheme, which is a non-trivial challenge^16,17,86^. Once successful, this could increase acceptance of ambitious market-based mitigation policies, such as a tax on non-CO_2_ emissions or emission trading scheme^17^, as it helps farmers, similar as other redistribution measures^87^, to compensate for part of the additional costs incurred through the adoption of a GHG pricing scheme^20,88–90^. Such policy incentives need to be designed in a way that ensures that the carbon remains in agricultural soils and biomass in the long run and agricultural practices are maintained once carbon accumulation saturates.

## Methods

### GLOBIOM–G4M

GLOBIOM^91^ is a global recursive dynamic partial equilibrium model of the forest and agricultural sectors and has been used extensively in different land-based mitigation assessments and for the representation of the land-use sectors in IAMs. It maximizes global producer and consumer surplus of agriculture and forestry calculating market equilibrium, bilateral trade-flows, spatially explicit land use and land-use changes, prices, GHG emissions and other economic and environmental variables. Commodity markets and international trade are represented at the level of 37 economic regions in this study. The spatial resolution of the supply side relies on the concept of simulation units, which are aggregates of 5–30 arcmin pixels belonging to the same altitude, slope and soil class and also the same country^92^. For crops, livestock and forest products, spatially explicit Leontief production functions covering alternative production systems are parameterized using bio-physical models such as the Environmental Policy Integrated Model^93^, G4M^41,94^ or the RUMINANT model^58^. The model includes six land cover types: cropland, grassland, short rotation plantations, managed forests, unmanaged forests and other natural vegetation land. Depending on the profitability of primary products, byproducts and final products production activities, the model can switch from one land cover type to another. GLOBIOM is linked with the G4M model^41,42^ for the detailed representation of forest management and carbon flows and an energy system model MESSAGEix^57,95^ for the interactions with the energy system and economy-wide impacts.

### Scenarios

Next to our baseline scenario that is based on the Shared Socioeconomic Pathway 2 (SSP2)^46^ under historical climate, we implement different land-based mitigation scenarios to assess the cost-effective mitigation potential of agricultural CO_2_ sequestration options. Main elements of the quantified mitigation scenarios are different GHG price trajectories on AFOLU emissions/removals and alternative bioenergy demand trajectories. We simulate a linearly increasing AFOLU GHG price from 2030 onwards that reaches 25, 50, 75, 100, 125, 150, 175 and 200 USD_2000_ tCO_2_e^−1^ by 2050. GHG prices were converted ex post from USD_2000_ to USD_2022_, applying a global uniform conversion rate of 1.63 using the US GDP deflator from the World Bank. This simplified approach does not capture differences in regional macro-economic developments. However, the proportional scaling ensures consistency of the presented results with the underlying partial equilibrium modelling performed in constant USD_2000_. Non-CO_2_ gases were converted to CO_2_ equivalents using global warming potentials from the 4th IPCC Assessment Report (298 for N_2_O and 25 for CH_4_). In the mitigation scenarios that do not consider agricultural CO_2_ sequestration options (‘default’ and ‘default_bio’), this GHG price is only included on agricultural non-CO_2_ emissions and FOLU CO_2_ emissions/removals, while in ‘agCO_2_’ and ‘agCO_2__bio’ the GHG price is also applied to agricultural CO_2_ removals. Bioenergy demand is either kept at baseline levels or at levels compatible with the 1.5 °C target (Table 1).

### Agricultural carbon sequestration options and crop residues

In this study, three carbon sequestration options on agricultural land were included:

1. Silvo-pasture systems for biomass and biochar production or carbon sequestration
2. Carbon sequestration through improved cropland and pasture management
3. Biochar application on cropland

To represent the new mitigation technologies, we applied a similar approach as described in Frank, Havlík^20^ and introduced explicit CO_2_ mitigation technologies on agricultural land using information on carbon sequestration coefficients per technology, economic costs, as well as information on the potential impact on crop and pasture productivities. Consequently, a marginal abatement cost curve can be emulated from the model by applying a GHG price, which triggers the adoption of mitigation technologies if the expected revenues, for example, through the avoided GHG price payments or improved productivities, exceed the costs of adoption of a given technology. The different CO_2_ options are assumed to be additive and can be adopted jointly on a piece of cropland (SOC and biochar) or pastures (SOC and silvo-pastures) in the model.

### Silvo-pasture systems

#### 3-PGmix model

3-PGmix is a simplified process-based forest growth model that uses a big-leaf approach to simulate stands dynamics. Moreover, 3-PGmix expands the original 3PG model^96^ by including modified processes for light interception, canopy transpiration and additional model features, enabling to simulate mixed forest stands and more complex canopy configurations^97^. The model operates in a monthly time step and simulates GPP using a light use efficiency approach, which considers multiple environmental drivers, including temperature, vapour pressure deficit, available soil water, soil fertility, number of frost days, atmospheric CO_2_ concentration and stand age, as well as absorbed photosynthetically active radiation and the canopy quantum efficiency. Net primary production is then calculated as a constant fraction of gross primary production. Subsequently, carbon is allocated to different tree compartments (roots, foliage and stem). The allocation to roots is prioritized, where harsher growing conditions induce a higher allocation of carbon to roots, and the allocation to foliage and roots follows from the remaining net primary production fraction, maintaining a balance between the growth rates of foliage and stem. Besides the dynamics related to the different biomass compartments, the model allows to derive several attributes relevant to management, including stand diameter at breast height, volume, basal area and mean annual increment, among others, based on allometric relationships^98^.

Tree plantations in silvo-pasture systems were assumed to be fertilized, hence, with no nutrient limitations. The fertilization demand was computed based on the available soil nitrogen and nitrogen demand from the plantations. To account from the available soil nitrogen, we have coupled the 3-PGmix model with the Yasso20 soil model^99^ and derived the nitrogen dynamics in the soil with the help of stochiometric relationships on the decomposition of various SOC compartments^100^. The nitrogen fertilization amounts were defined based on the increment of the biomass compartments in the plantations and the respective nitrogen concentration in plant tissues. Phosphorus demand was established as a constant fraction of the nitrogen demand.

#### Simulation setup

The productive potentials were computed in the model, using the GLOBIOM 5 to 30 arcmin simulation units^92^, where typical growing conditions were defined. Soil inputs to the model (maximum available soil water, soil texture, carbon and nitrogen stocks) were retrieved from the ISRIC soil database^101^. Climate inputs (minimum temperature, maximum temperature, mean temperature, precipitation, solar radiation and number of forest days) were computed for each simulation unit for historic climate based on the WorldClim version 2.1 data^102^.

For each simulation unit, we selected the appropriate species based on the climate attributes. Plantations were primarily composed by different Eucalypt species, including *Eucalyptus* *saligna*, *Eucalyptus* *pellitta*, *Eucalyptus* *grandis*, *Eucalyptus* *urophylla* and *Eucalyptus* *globulus*, as well as poplar (*Populus spp*.), depending on the climate attributes, specifically temperature and precipitation regimes, based on Booth^103^. For grid cells in temperate and boreal ecosystems not suitable for *E. globulus* (mean annual temperature below 11 °C), poplar plantations were established. The parameters for each species were retrieved from the 3-PGmix parameter database, contained in the R package r3PG^104^.

#### Silvo-pasture representation in GLOBIOM

Two explicit silvo-pasture systems^105–110^ were implemented in GLOBIOM based on the bio-physical 3-PGmix simulation.

##### Silvo-pastures for bioenergy and biochar production

The 3-PGmix model was used to simulate productivities, carbon sequestration in above- and belowground biomass (Supplementary Table 3) and nitrogen inputs of short rotation tree plantations for a 10 year rotation period, consistent with GLOBIOM internal logic. These data were combined with pasture productivities in GLOBIOM^13^ assuming that 25% of the pasture area would be planted in alleys with short rotation tree plantations^110,111^ and harvested in a 10 year rotation, which corresponds to approximately 1,250–2,500 trees per hectare, depending on the species, with higher density for poplar plantations. Harvested biomass from short rotation tree plantations can be used for either bioenergy or biochar production in the model. In this system, the new equilibrium in biomass carbon stocks is assumed to be reached after 10 years following the establishment. Costs for the establishment, maintenance and harvest of short rotation tree plantations are based on Havlík et al.^112^.

##### Silvo-pasture for carbon sequestration

The 3-PGmix model was used to simulate productivities, carbon sequestration in above- and belowground biomass (Supplementary Table 3) and nitrogen inputs of fast-growing tree species for a 30 year rotation period. As for silvo-pasture system for biomass production, 25% of the pasture area was assumed to be planted with trees. Given the longer rotation period of 30 years, this results in a lower tree density of around 400–600 trees per hectare, depending on the species, but higher biomass accumulation over a longer rotation period. Accumulation of carbon in biomass is assumed to continue over a 30 year period. Short rotation tree plantations costs based on ref. ^112^ were decomposed to account only for establishment and maintenance costs that were calculated using a bottom-up costing approach^113,114^. Owing to the longer rotation time, the reduced planting density, limited maintenance and no harvesting costs, this system is much cheaper with only 8% (on global average) of the total costs of the silvo-pasture system for bioenergy production.

In both systems, we applied the conservative assumption of no pasture productivity increases due to efficiency gains in response to the conversion to silvo-pasture system. Hence, grazing biomass supply declines by 25% to account for the planting of trees on 25% of the area. Adoption of silvo-pasture systems was limited to 50% of the total pasture area in a region.

### Enhanced SOC sequestration on cropland and pastures

Annualized carbon sequestration coefficients at the country level over the 2020–2050 period are based on Roe et al.^25^ for cropland and pastures. Sequestration rates (Supplementary Table 3) are assumed not to change dynamically over time, and a saturation of the carbon sequestration potential is assumed after 20 years in line with IPCC guidelines^115^. Associated yield increases for the improvement of cropland SOC on degraded land following Smith et al.^116^ have been implemented for Africa, Latin America and Asia based data from Lal^117^. Annual yield increases of crop aggregates reached 1.5%, 1.2% and 0.7% in Africa, Latin America and Asia, respectively, and 0.9% at world average, per tCO_2_ ha^−1^ sequestered annually.

A quadratic cost function was implemented to calibrate the adoption rates of these mitigation technologies in GLOBIOM. The slope of the cropland and pasture cost curve was fitted to approximate adoption rates (90% for cropland and 60% for grassland) at a carbon price of 100 USD_2000_ tCO_2_^−1^ as presented in Roe et al.^25^. The maximum adoption potential of 90% of cropland area and 60% of pastures was assumed following Roe et al.^25^. Improved cropland management can be combined with other mitigation options on the same spot of land, such as improved fertilization or biochar application. Improved pasture management can be combined with silvo-pasture systems.

### Biochar application

Emission factors (Supplementary Table 3) for biochar application on cropland are based on the annualized (2020–2050 period) data from Roe et al.^25^ and assuming saturation of the carbon sequestration potential following 30 years of application. Crop yield improvement from biochar application were calculated using the carbon sequestration coefficients and applying the method, as done for improved crop- and grassland management options following Lal^117^. Costs for pyrolysis, storage and processing and application to land of 35 USD_2000_ tCO_2_e^−1^ were based on Homagain et al.^118^. Conversion factors for biochar production were based on Griscom et al.^24^ assuming 0.45% carbon content per ton biomass input, 50% of which is retained and 79.6% stored for more than 100 years in biochar once applied to the soil, which yields a conversion efficiency of 0.18 tCe biochar per tdm biomass input. In the model, biochar competes for biomass with other energy and material uses^119^ and can be produced from crop residues, logging residues, bark, wood chips, recycled wood or short rotation coppices/tree plantations. A total of 50% of the biomass feedstock was assumed to be available for bioenergy production during the pyrolysis process as byproduct following Wang et al.^32^.

On the supply side (Supplementary Table 4), the technical crop residue potential was parameterized in GLOBIOM using endogenous crop productivity estimates and applying crop specific residue-product ratios (except for oil palm) from Holmatov et al.^120^. It was assumed that 50% of the technical potential could be sustainable removed^121^ without impacts on crop yields and SOC stocks. Costs for crop residue baling, recovery and transportation were based on the BMLFUW^122^ and rescaled across world regions using GDP per capita differences. Secondary crop residues from processing were not considered for bioenergy or biochar production. On the demand side, crop residue demand for livestock production (occasional feeding and bedding) is based on the coefficients from Herrero et al.^58^. In our baseline, we assume that 50% of the other (non-forest) solid biomass demand is sourced from crop residues (~16 EJ per year in 2020). Overall, crop residue demand competes across the different uses in the model (livestock, bioenergy and biochar production).

### Economic impact on farmers

To assess the economic impact of a GHG price on farmers, an ex post calculation was performed. The results presented in Fig. 4 show changes of three mitigation scenarios compared with the baseline scenario without mitigation efforts. Changes in gross turnover for crop and livestock products were calculated by multiplying differences in production quantities and prices of agricultural products when comparing the mitigation scenarios with the baseline in 2050. Positive values indicate an increase in gross turnover for producers. GHG tax payments were calculated by multiplying agricultural GHG emissions with the GHG price. Tax payments were shown as negative values indicating a cost for producers. Carbon subsidy payments were calculated by multiplying CO_2_ removals (sequestration) on agricultural land with the GHG price. Carbon subsidy payments were shown as positive value indicating a payment to producers. The total net turnover effect for producers was calculated by summing up gross turnover changes (typically positive), carbon subsidy revenues (positive) and GHG tax payments (negative).

To assess impacts on producers and government budgets, the data were rearranged in Fig. 4b. Effects on producers are equivalent to net turnover effect (gross turnover, GHG tax payments and carbon subsidy revenues). The impact on government budget is calculated by summing up GHG tax and carbon subsidy payments. Unlike in Fig. 4a, the sign is different as the GHG tax represents a payment for producers (negative) but an income for the government (positive). Hence, a negative value in Fig. 4b for the government indicates that carbon subsidy payments exceed the GHG tax revenues the government receives.

### Reporting summary

Further information on research design is available in the Nature Portfolio Reporting Summary linked to this article.

## Supplementary information

Supplementary Information Supplementary information with additional information on the applied modelling framework.

Reporting Summary

Supplementary Data 1 Dataset containing GLOBIOM model results.

## Data Availability

The results that support the findings of the study are provided in the paper and in Supplementary Information. The sources of all data used in this study are referenced in Methods.

## Code availability

A GitHub repository at https://iiasa.github.io/GLOBIOM/introduction.html provides GLOBIOM documentation, links to GLOBIOM resources, GAMS script descriptions and dependency links that match the Trunk version of the GLOBIOM. Additional code can be made available upon request from the corresponding author.
