# Supplementary material for: Enhanced agricultural carbon sinks provide benefits for farmers and the climate
Source: Nat Food. 2024 Sep 23;5(9):742–53. doi: 10.1038/s43016-024-01039-1 (PMC11420069; doi:10.1038/s43016-024-01039-1)
Supplement: Supplementary file 1 — Supplementary information with additional information on the applied modelling framework. [file 43016_2024_1039_MOESM1_ESM.pdf]

nature food

Supplementary information https://doi.org/10.1038/s43016-024-01039-1

# Enhanced agricultural carbon sinks provide benefits for farmers and the climate

In the format provided by the authors and unedited

# Table of Contents

1 Modelling framework ..... 2
1.1 GLOBIOM-G4M ..... 2
1.2 Scenario development ..... 4
1.2.1 Baseline ..... 4
1.2.2 AFOLU sector GHG mitigation scenarios ..... 4
1.2.3 Economy-wide climate stabilization scenarios ..... 5
2 Additional parameters ..... 7
3 References ..... 11

# 1 Modelling framework

## 1.1 GLOBIOM-G4M

The Global Biosphere Management Model (GLOBIOM) (IBF-IIASA 2023) is a partial equilibrium model that covers the global agricultural and forestry sectors, including the bioenergy sector. Commodity markets and international trade are represented at the level of 37 economic regions in this study. Prices are endogenously determined at the regional level to establish market equilibrium to reconcile demand, domestic supply and international trade. The spatial resolution of the supply side relies on the concept of Simulation Units, which are aggregates of 5 to 30 arcmin pixels belonging to the same altitude, slope, and soil class, and also the same country (Skalský et al. 2008). For crops, livestock, and forest products, spatially explicit Leontief production functions covering alternative production systems are parameterized using biophysical models like EPIC (Environmental Policy Integrated Model) (Williams 1995), G4M (Global Forest Model) (Kindermann, McCallum, et al. 2008; Gusti 2010), or the RUMINANT model (Herrero et al. 2013). For the present study, the supply side spatial resolution was aggregated to 2 degrees (about 200 x 200 km at the equator). Land and other resources are allocated to the different production and processing activities to maximize a social welfare function which consists of the sum of producer and consumer surplus. The model includes six land cover types: cropland, grassland, short rotation plantations, managed forests, unmanaged forests, and other natural vegetation land. Depending on the relative profitability of primary, by-, and final products production activities, the model can switch from one land cover type to another. Spatially explicit land conversion at the simulation unit level over the simulation period is endogenously determined within the available land resources and conversion costs. Land conversion cost parameters are region specific to match observed land use changes. Land conversion possibilities are further restricted through biophysical land suitability and production potentials, and through a matrix of potential land cover transitions. Land conversion from one land cover to the other takes place if the marginal revenue of the new production activity exceeds the marginal land conversion cost. GLOBIOM covers major GHG emissions from agricultural production, forestry, and other land use including $CO_2$ emissions from above- and belowground biomass changes, $N_2O$ from the application of synthetic fertilizer and manure to soils, $N_2O$ from manure dropped on pastures, $CH_4$ from rice cultivation, $N_2O$ and $CH_4$ from manure management, and $CH_4$ from enteric fermentation. $CO_2$ emissions/removals from afforestation, deforestation, and wood production in managed forests are estimated by geographically explicit (0.5x0.5 degree) model G4M (Kindermann, Obersteiner, et al. 2008; Gusti 2010) that is connected with GLOBIOM. Afforestation and deforestation decisions are calculated by comparing net present values of agriculture and forestry land uses. Afforestation occurs where it is more profitable than the agriculture and the environmental conditions are suitable for forest growth. Deforestation, in contrast, happens where agriculture net present value plus profit from one-time selling of deforested wood

exceeds the net present value of forestry. The net present values are estimated considering agriculture land rents and wood prices obtained from GLOBIOM and price of carbon stored in biomass. The land transitions in G4M are harmonized with GLOBIOM agriculture land demand. G4M simulates forest management aimed at sustainable production of wood demanded by GLOBIOM at regional scale.

In Table S1 we refer to relevant references for detailed information on the main model structure, datasets used, or individual modules.

*Table S1. Key references for model documentation.*

| **Topic** | **Sub-topic** | **Brief description** | **Key references** |
|---|---|---|---|
| GLOBIOM model | Model overview | Model overview, structure, equations, and datasets used | Havlík et al. (2014), Havlík et al. (2011), Valin et al. (2013) |
| | Crop- and livestock production | Representation of crop- and livestock production systems | Havlík et al. (2014) |
| | Forestry and biomass production | Forest sector and forest industry representation, biomass feedstocks and potentials | Lauri et al. (2017), Lauri et al. (2019) |
| | Food consumption and demand | Representation of food demand and projections | Valin et al. (2014) |
| | International trade | International trade representation | Janssens et al. (2020) |
| G4M model | Model overview | Model overview, structure, equations, and datasets used | Kindermann, Obersteiner, et al. (2008), Gusti (2010) |
| | GLOBIOM-G4M | Link to GLOBIOM for AFOLU climate change mitigation assessments | Frank et al. (2021), Lauri et al. (2017) |
| MESSAGEix-GLOBIOM | Model overview | Model overview, structure, equations, and datasets used | Krey et al. (2020), Huppmann et al. (2019) |
| Land-based mitigation potentials | Agriculture MACCs | Agricultural non-$CO_2$ mitigation potentials | Frank et al. (2018), Frank et al. (2019) |
| | FOLU MACCs | Forestry and other land use (FOLU) $CO_2$ mitigation potentials | Frank et al. (2021) |
| Scenario development | Shared Socio-Economic Pathways | SSP scenario implementation in GLOBIOM-G4M and link to MESSAGEix | Fricko et al. (2017) |
| | Climate stabilization pathways | 1.5 °C stabilization pathways using MESSAGEix-GLOBIOM, land-based mitigation requirements (carbon prices, bioenergy demands) | Rogelj et al. (2018), Hasegawa et al. (2021) |

## 1.2 Scenario development

### 1.2.1 Baseline

The baseline scenario corresponds to the SSP2 middle-of-the-road scenario without land-based climate mitigation efforts (Fricko et al. 2017). Population and GDP projections were implemented in GLOBIOM based on the SSP database (https://tntcat.iiasa.ac.at/SspDb/). Income elasticities are calibrated to mimic FAO projections of diets (Alexandratos and Bruinsma 2012). We assume moderate reductions in food waste and losses over time add to the availability of agricultural products (FAO 2011). Technological change for crops is based on crop specific yield response functions to GDP per capita growth estimated for different income groups using a fixed effects model (Havlík et al. 2014). Fertilizer use and costs of agricultural production increase in proportion with yields. Improvements in livestock feed conversion efficiencies follow Bouwman et al. (2005). Transition towards more efficient livestock production systems takes place at a moderately fast pace. Biomass demand for bioenergy is projected to remain rather stable at around 60 EJ/yr until mid- century based on MESSAGE-GLOBIOM projections. Details on the SSP drivers and scenario implementation are provided in (Fricko et al. 2017).

### 1.2.2 AFOLU sector GHG mitigation scenarios

On top of the baseline, we implemented eight climate change mitigation scenarios differentiated by a GHG price trajectory on agriculture, forestry, and other land use (AFOLU) emissions/removals. The GHG prices were implemented as off 2020 as an additional cost for emissions (or subsidy for removals) reaching a GHG price of 25, 50, 75, 100, 125, 150, 175, and 200 $USD_{2000}/tCO_2e$ by 2050. GHG prices were converted ex-post from $USD_{2000}$ to $USD_{2022}$ applying a global uniform conversion rate of 1.63 using the US GDP deflator from the World Bank. This simplified approach does not capture differences in regional macro-economic developments. However, the proportional scaling ensures consistency of the presented results with the underlying partial equilibrium modelling framework which was performed in constant $USD_{2000}$. Non-$CO_2$ gases were converted to $CO_2$ equivalents ($CO_2e$) using global warming potentials from the 4$^{th}$ IPCC Assessment Report (298 for $N_2O$, 25 for $CH_4$). The following gases were included under the GHG price:

- Agricultural $N_2O$ emissions: synthetic fertilizer, manure applied to soils and dropped on pastures, manure management
- Agricultural $CH_4$ emissions: rice cultivation, enteric fermentation, manure management
- Agricultural $CO_2$ removals: soil carbon sequestration from improved cropland and grassland management, above- and belowground biomass carbon sequestration from silvo-pasture systems, emission reduction (mainly $CO_2$) from biochar application on cropland
- Forestry $CO_2$ emissions/removals: afforestation, deforestation, forest management, and other land use changes

Emissions from organic soils, agricultural residue burning, and savannah burning were assumed to be kept constant over time.

*Table S2. Carbon price and bioenergy demand trajectories over time.*

| | | 2020 | 2030 | 2040 | 2050 | 2060 | 2070 |
|---|---|---|---|---|---|---|---|
| GHG price [$USD_{2000}/tCO_2e$] | Baseline | 0 | 0 | 0 | 0 | 0 | 0 |
| | GHG price path 1 | 0 | 8 | 17 | 25 | 33 | 42 |
| | GHG price path 2 | 0 | 17 | 33 | 50 | 67 | 83 |
| | GHG price path 3 | 0 | 25 | 50 | 75 | 100 | 125 |
| | GHG price path 4 | 0 | 33 | 67 | 100 | 133 | 167 |
| | GHG price path 5 | 0 | 42 | 83 | 125 | 167 | 208 |
| | GHG price path 6 | 0 | 50 | 100 | 150 | 200 | 250 |
| | GHG price path 7 | 0 | 58 | 117 | 175 | 233 | 292 |
| | GHG price path 8 | 0 | 67 | 133 | 200 | 267 | 333 |
| Bioenergy demand [EJ/yr] | Baseline | 57 | 60 | 59 | 60 | 63 | 65 |
| | 1.5 °C scenario | 57 | 72 | 86 | 115 | 147 | 174 |

### 1.2.3 Economy-wide climate stabilization scenarios

To assess the impact of agricultural $CO_2$ sequestration options on the economy-wide mitigation portfolio, we quantified two 1.5 °C mitigation scenarios in MESSAGEix-GLOBIOM (Fricko et al. 2017; Krey et al. 2020) with- and without considering agricultural $CO_2$ sequestration options in the AFOLU mitigation potentials. MESSAGEix (Model for Energy Supply Strategy Alternatives and their General Environmental Impact), is a linear programming system engineering model used for medium- to long-term energy system planning, energy policy analysis, and scenario development (Huppmann et al. 2019; Messner and Strubegger 1995). The model provides a framework for representing an energy system with all its interdependencies from resource extraction, imports and exports, conversion, transport, and distribution, to the provision of energy end-use services such as light, space conditioning, industrial production processes, and transportation. MESSAGEix is linked to GLOBIOM to assess the implications of utilizing bioenergy of different types and to integrate the GHG emissions from energy and land use (Fricko et al. 2017) and to the aggregated single-sector macro-economic model MACRO to assess economic implications and economy-wide feedbacks (Messner and Schrattenholzer 2000). MACRO is a macroeconomic model maximizing the intertemporal utility function of a single representative producer-consumer in each world region and is used to generate a consistent economic

response (e.g., changes in GDP or household consumption) to changes in energy prices (from MESSAGEix) because of energy or climate policies.

For this study, we quantified two 1.5 °C scenario based on the EN_NPi2020_600 no-overshoot scenario with a remaining carbon budget of 600 $GtCO_2$ from the ENGAGE project (Riahi et al. 2021; Hasegawa et al. 2021) in MESSAGEix-GLOBIOM that considered or did not consider $CO_2$ sequestration options on agricultural land in the AFOLU mitigation potentials.

# 2 Additional parameters

*Table S3. Annual sequestration coefficients across agricultural $CO_2$ sequestration technologies in $tCO_2/ha$.*

| | Cropland Mgmt SOC | Biochar application | Silvopasture $CO_2$ sequestration | Silvopasture bioenergy/biochar | Pasture Mgmt SOC |
|---|---|---|---|---|---|
| Argentina | -0.39 | -2.74 | -1.77 | -2.05 | -0.39 |
| Australia | -0.20 | -1.54 | -1.17 | -1.56 | -0.29 |
| Brazil | -1.21 | -4.86 | -0.99 | -1.80 | -0.79 |
| Canada | -0.74 | -1.43 | -0.97 | -0.94 | -1.15 |
| China | -0.86 | -4.38 | -1.39 | -1.74 | -0.99 |
| Congo Basin | -1.22 | -0.64 | -0.90 | -1.83 | -0.98 |
| EU Baltic | -2.51 | -1.20 | -1.27 | -1.06 | -3.75 |
| EU Central East | -0.41 | -1.95 | -1.16 | -1.00 | -2.04 |
| EU Central West | -0.65 | -1.76 | -1.40 | -1.27 | -1.13 |
| EU North | -1.95 | -1.22 | -1.58 | -1.39 | -1.95 |
| EU South | -0.32 | -1.73 | -1.34 | -1.41 | -0.90 |
| Former USSR | -0.44 | -0.90 | -1.16 | -1.07 | -0.76 |
| India | -0.90 | -1.62 | -1.48 | -2.07 | -1.71 |
| Indonesia | -0.49 | -3.78 | -1.65 | -2.76 | -1.76 |
| Japan | -2.74 | -5.11 | -1.59 | -1.61 | -1.77 |
| Malaysia | -0.36 | -2.93 | -1.82 | -3.27 | -2.97 |
| Mexico | -0.58 | -3.14 | -1.13 | -1.76 | -0.99 |
| Middle East | -0.25 | -1.01 | -0.73 | -1.20 | -0.63 |
| New Zealand | -1.20 | -2.15 | -1.78 | -1.98 | -1.46 |
| Northern Africa | -0.26 | -1.46 | - | - | -0.62 |
| Pacific Islands | -0.85 | -1.71 | -2.06 | -3.07 | -1.38 |
| RCAM | -1.26 | -3.98 | -1.45 | -2.48 | -1.98 |
| RCEU | -0.58 | -2.55 | -1.18 | -1.05 | -2.64 |
| ROWE | -1.88 | -1.28 | -1.68 | -1.51 | -2.58 |
| RSAM | -1.25 | -3.26 | -1.18 | -2.06 | -0.99 |
| RSAS | -0.80 | -2.71 | -1.44 | -1.94 | -0.78 |
| RSEA OPA | -1.08 | -3.66 | -1.51 | -2.19 | -1.98 |

| | | | | | |
|---|---|---|---|---|---|
| RSEA PAC | -1.16 | -4.51 | -1.48 | -1.87 | -1.04 |
| Russian Federation | -0.55 | -1.08 | -1.17 | -1.01 | -1.32 |
| South Africa | -0.31 | -2.00 | -1.59 | -1.95 | -0.88 |
| South Korea | -2.11 | -4.86 | -1.44 | -1.72 | -2.40 |
| Eastern Africa | -0.69 | -0.87 | -1.60 | -2.05 | -0.75 |
| Southern Africa | -0.50 | -0.79 | -1.28 | -1.85 | -0.84 |
| Western Africa | -0.49 | -0.53 | -0.85 | -1.76 | -0.67 |
| Turkey | -0.29 | -1.19 | -0.93 | -1.02 | -1.43 |
| Ukraine | -0.36 | -1.85 | -1.14 | -0.93 | -1.73 |
| United States | -0.70 | -3.43 | -1.19 | -1.35 | -0.82 |

*Table S4. Global crop residue potential.*

| | **Crop yield [t/ha]** | **Dry matter content [%]** | **Crop Residue - Production Ratio** | **Sustainable removal rate [%]** | **Residue yield [t/ha]** |
|---|---|---|---|---|---|
| Barley | 2.5 | 15 | 1.7 | 50 | 1.8 |
| Beans dry | 0.7 | 10 | 2.5 | 50 | 0.8 |
| Cassava | 10.4 | 65 | 0.2 | 50 | 0.4 |
| Chickpeas | 0.8 | 10 | 2.5 | 50 | 0.9 |
| Groundnut | 1.5 | 15 | 2.5 | 50 | 1.6 |
| Corn | 4.4 | 15 | 1.5 | 50 | 2.8 |
| Millet | 0.8 | 15 | 3.0 | 50 | 1.0 |
| Potatoes | 16.0 | 65 | 0.8 | 50 | 2.1 |
| Rapeseed | 1.5 | 40 | 2.8 | 50 | 1.2 |
| Rice | 3.9 | 15 | 1.5 | 50 | 2.5 |
| Cotton | 1.0 | 15 | 3.5 | 50 | 1.5 |
| Sorghum | 1.4 | 15 | 2.6 | 50 | 1.5 |
| Soya | 2.3 | 15 | 2.5 | 50 | 2.4 |
| Sugarcane | 64.6 | 75 | 0.3 | 50 | 2.4 |
| Sunflower | 1.2 | 40 | 2.6 | 50 | 1.0 |
| Sweet potatoes | 14.6 | 65 | 0.6 | 50 | 1.5 |
| Wheat | 2.7 | 15 | 1.2 | 50 | 1.4 |

*Table S5. Regional mapping of countries.*

| Aggregates | GLOBIOM region | Country |
|---|---|---|
| CIS | Former USSR | Armenia, Azerbaijan, Belarus, Georgia, Kazakhstan, Kyrgyzstan, Moldova, Tajikistan, Turkmenistan, Uzbekistan |
| | Russian Federation | Russian Federation |
| | Ukraine | Ukraine |
| EAS | China | China |
| | Japan | Japan |
| | South Korea | South Korea |
| EUR | EU Baltic<br>EU Central East<br>EU Central West<br>EU North<br>EU South | Austria, Belgium, Bulgaria, Croatia, Cyprus, Czech Republic, Denmark, Estonia, Finland, France, Germany, Greece, Hungary, Ireland, Italy, Latvia, Lithuania, Luxembourg, Malta, Netherlands, Poland, Portugal, Romania, Slovakia, Slovenia, Spain, Sweden, UK |
| | RCEU | Albania, Bosnia and Herzegovina, Macedonia, Serbia-Montenegro |
| | ROWE | Iceland, Norway, Switzerland |
| SAM | Argentina | Argentina |
| | Brazil | Brazil |
| | RSAM | Bolivia, Chile, Colombia, Ecuador, Guyana, Paraguay, Peru, Suriname, Uruguay, Venezuela |
| MAF | Middle East | Bahrain, Iran, Iraq, Israel, Jordan, Kuwait, Lebanon, Oman, Qatar, Saudi Arabia, Syria, United Arab Emirates, Yemen |
| | Northern Africa | Algeria, Egypt, Libya, Morocco, Tunisia, Western Sahara |
| | Turkey | Turkey |
| NAM | Canada | Canada |
| | Mexico | Mexico |
| | RCAM | Bahamas, Belize, Costa Rica, Cuba, Dominican Republic, El Salvador, Guatemala, Haiti, Honduras, Jamaica, Nicaragua, Panama, Trinidad and Tobago |
| | United States | United States |
| OCE | Australia | Australia |
| | New Zealand | New Zealand |
| | Pacific Islands | Fiji Islands, Papua New Guinea, Samoa, Solomon Islands, Vanuatu |
| SAS | India | India |
| | RSAS | Bangladesh, Bhutan, Nepal, Pakistan, Sri Lanka |
| SEA | Indonesia | Indonesia |
| | Malaysia | Malaysia |
| | RSEA OPA | Brunei Darussalam, Myanmar, Philippines, Singapore, Thailand |
| | RSEA PAC | Cambodia, Korea DPR, Laos, Mongolia, Vietnam |

| | | |
|---|---|---|
| SSA | Congo Basin | Cameroon, Central African Republic, Congo Republic, Democratic Republic of Congo, Equatorial Guinea, Gabon |
| | Eastern Africa | Burundi, Ethiopia, Kenya, Rwanda, Tanzania, Uganda |
| | Southern Africa | Angola, Botswana, Comoros, Lesotho, Madagascar, Malawi, Mauritius, Mozambique, Namibia, Swaziland, Zambia, Zimbabwe |
| | Western Africa | Benin, Burkina Faso, Cape Verde, Chad, Cote d'Ivoire, Djibouti, Eritrea, Gambia, Ghana, Guinea, Guinea Bissau, Liberia, Mali, Mauritania, Niger, Nigeria, Senegal, Sierra Leone, Somalia, Sudan, Togo |
| | South Africa | South Africa |

# 3 References


Alexandratos, N., and J. Bruinsma. 2012. "World Agriculture Towards 2030/2050 The 2012 Revision." In, 160. Rome: FAO.

Bouwman, A. F., K. W. Van Der Hoek, B. Eickhout, and I. Soenario. 2005. 'Exploring changes in world ruminant production systems', *Agricultural Systems*, 84: 121-53.

FAO. 2011. "Global food losses and food waste: Extent, causes, and prevention." In, 38. Rome: FAO.

Frank, S., R. Beach, P. Havlík, H. Valin, M. Herrero, A. Mosnier, T. Hasegawa, J. Creason, S. Ragnauth, and M. Obersteiner. 2018. 'Structural change as a key component for agricultural non-CO2 mitigation efforts', *Nature Communications*, 9: 1060.

Frank, Stefan, Mykola Gusti, Petr Havlík, Pekka Lauri, Fulvio DiFulvio, Nicklas Forsell, Tomoko Hasegawa, Tamás Krisztin, Amanda Palazzo, and Hugo Valin. 2021. 'Land-based climate change mitigation potentials within the agenda for sustainable development', *Environmental Research Letters*, 16: 024006.

Frank, Stefan, Petr Havlík, Elke Stehfest, Hans van Meijl, Peter Witzke, Ignacio Pérez-Domínguez, Michiel van Dijk, Jonathan C. Doelman, Thomas Fellmann, Jason F. L. Koopman, Andrzej Tabeau, and Hugo Valin. 2019. 'Agricultural non-CO2 emission reduction potential in the context of the 1.5 °C target', *nature climate change*, 9: 66-72.

Fricko, O., P. Havlik, J. Rogelj, Z. Klimont, M. Gusti, N. Johnson, P. Kolp, M. Strubegger, H. Valin, M. Amann, T. Ermolieva, N. Forsell, M. Herrero, C. Heyes, G. Kindermann, V. Krey, D. L. McCollum, M. Obersteiner, S. Pachauri, S. Rao, E. Schmid, W. Schoepp, and K. Riahi. 2017. 'The marker quantification of the Shared Socioeconomic Pathway 2: A middle-of-the-road scenario for the 21st century', *Global Environmental Change*, 42: 251-67.

Gusti, M. 2010. 'An algorithm for simulation of forest management decisions in the global forest model', *Artificial Intelligence*, N4: 45-49.

Hasegawa, Tomoko, Shinichiro Fujimori, Stefan Frank, Florian Humpenöder, Christoph Bertram, Jacques Després, Laurent Drouet, Johannes Emmerling, Mykola Gusti, Mathijs Harmsen, Kimon Keramidas, Yuki Ochi, Ken Oshiro, Pedro Rochedo, Bas van Ruijven, Anique-Marie Cabardos, Andre Deppermann, Florian Fosse, Petr Havlik, Volker Krey, Alexander Popp, Roberto Schaeffer, Detlef van Vuuren, and Keywan Riahi. 2021. 'Land-based implications of early climate actions without global net-negative emissions', *Nature Sustainability*.

Havlík, Petr, Uwe A. Schneider, Erwin Schmid, Hannes Böttcher, Steffen Fritz, Rastislav Skalský, Kentaro Aoki, Stéphane De Cara, Georg Kindermann, Florian Kraxner, Sylvain Leduc, Ian McCallum, Aline Mosnier, Timm Sauer, and Michael Obersteiner. 2011. 'Global land-use implications of first and second generation biofuel targets', *Energy Policy*, 39: 5690-702.

Havlík, Petr, Hugo Valin, Mario Herrero, Michael Obersteiner, Erwin Schmid, Mariana C. Rufino, Aline Mosnier, Philip K. Thornton, Hannes Böttcher, Richard T. Conant, Stefan Frank, Steffen Fritz, Sabine Fuss, Florian Kraxner, and An Notenbaert. 2014. 'Climate change mitigation through livestock system transitions', *Proceedings of the National Academy of Sciences*, 111: 3709-14.

Herrero, Mario, Petr Havlík, Hugo Valin, An Notenbaert, Mariana C. Rufino, Philip K. Thornton, Michael Blümmel, Franz Weiss, Delia Grace, and Michael Obersteiner. 2013. 'Biomass use, production, feed efficiencies, and greenhouse gas emissions from global livestock systems', *Proceedings of the National Academy of Sciences*, 110: 20888-93.

Huppmann, Daniel, Matthew Gidden, Oliver Fricko, Peter Kolp, Clara Orthofer, Michael Pimmer, Nikolay Kushin, Adriano Vinca, Alessio Mastrucci, Keywan Riahi, and Volker Krey. 2019. 'The MESSAGEix Integrated Assessment Model and the ix modeling platform (ixmp): An open framework for integrated and cross-cutting analysis of energy, climate, the environment, and sustainable development', *Environmental Modelling & Software*, 112: 143-56.

IBF-IIASA. 2023. "Global Biosphere Management Model (GLOBIOM) Documentation 2023 - Version 1.0." In, 92. Laxenburg, Austria: International Institute for Applied Systems Analysis.

Janssens, Charlotte, Petr Havlík, Tamás Krisztin, Justin Baker, Stefan Frank, Tomoko Hasegawa, David Leclère, Sara Ohrel, Shaun Ragnauth, Erwin Schmid, Hugo Valin, Nicole Van Lipzig, and Miet Maertens. 2020. 'Global hunger and climate change adaptation through international trade', *nature climate change*, 10: 829-35.

Kindermann, G. E., I. McCallum, S. Fritz, and M. Obersteiner. 2008. 'A global forest growing stock, biomass and carbon map based on FAO statistics', *Silva Fennica*, 42: 387-96.

Kindermann, G., M. Obersteiner, B. Sohngen, J. Sathaye, K. Andrasko, E. Rametsteiner, B. Schlamadinger, S. Wunder, and R. Beach. 2008. 'Global cost estimates of reducing carbon emissions through avoided deforestation', *Proceedings of the National Academy of Sciences of the United States of America*, 105: 10302-07.

Krey, V., P. Havlik, P. N. Kishimoto, O. Fricko, J. Zilliacus, M. Gidden, M. Strubegger, G. Kartasasmita, T. Ermolieva, N. Forsell, M. Gusti, N. Johnson, J. Kikstra, G. Kindermann, P. Kolp, F. Lovat, D. L. McCollum, J. Min, S. Pachauri, S. C. Parkinson, S. Rao, J. Rogelj, G. Ünlü, H. Valin, P. Wagner, B. Zakeri, M. Obersteiner, and K. Riahi. 2020. "MESSAGEix-GLOBIOM Documentation." In. Laxenburg, Austria: International Institute for Applied Systems Analysis (IIASA).

Lauri, Pekka, Nicklas Forsell, Mykola Gusti, Anu Korosuo, Petr Havlík, and Michael Obersteiner. 2019. 'Global Woody Biomass Harvest Volumes and Forest Area Use Under Different SSP-RCP Scenarios', *Journal of Forest Economics*, 34: 285-309.

Lauri, Pekka, Nicklas Forsell, Anu Korosuo, Petr Havlík, Michael Obersteiner, and Annika Nordin. 2017. 'Impact of the 2°C target on global woody biomass use', *Forest Policy and Economics*, 83: 121-30.

Messner, S., and M. Strubegger. 1995. "User's Guide for MESSAGE III, WP-95-69." In.: International Institute for Applied Systems Analysis, Laxenburg, Austria.

Messner, Sabine, and Leo Schrattenholzer. 2000. 'MESSAGE–MACRO: linking an energy supply model with a macroeconomic module and solving it iteratively', *Energy*, 25: 267-82.

Riahi, Keywan, Christoph Bertram, Daniel Huppmann, Joeri Rogelj, Valentina Bosetti, Anique-Marie Cabardos, Andre Deppermann, Laurent Drouet, Stefan Frank, Oliver Fricko, Shinichiro Fujimori, Mathijs Harmsen, Tomoko Hasegawa, Volker Krey, Gunnar Luderer, Leonidas Paroussos, Roberto Schaeffer, Matthias Weitzel, Bob van der Zwaan, Zoi Vrontisi, Francesco Dalla Longa, Jacques Després, Florian Fosse, Kostas Fragkiadakis, Mykola Gusti, Florian Humpenöder, Kimon Keramidas, Paul Kishimoto, Elmar Kriegler, Malte Meinshausen, Larissa P. Nogueira, Ken Oshiro, Alexander Popp, Pedro R. R. Rochedo, Gamze Ünlü, Bas van Ruijven, Junya Takakura, Massimo Tavoni, Detlef van Vuuren, and Behnam Zakeri. 2021. 'Cost and attainability of meeting stringent climate targets without overshoot', *nature climate change*, 11: 1063-69.

Rogelj, Joeri, Alexander Popp, Katherine V. Calvin, Gunnar Luderer, Johannes Emmerling, David Gernaat, Shinichiro Fujimori, Jessica Strefler, Tomoko Hasegawa, Giacomo Marangoni, Volker Krey, Elmar Kriegler, Keywan Riahi, Detlef P. van Vuuren, Jonathan Doelman, Laurent Drouet, Jae Edmonds, Oliver Fricko, Mathijs Harmsen, Petr Havlík, Florian Humpenöder, Elke Stehfest, and Massimo Tavoni. 2018. 'Scenarios towards limiting global mean temperature increase below 1.5 °C', *nature climate change*, 8: 325-32.

Skalský, R., Z. Tarasovičová, J. Balkovič, E. Schmid, M. Fuchs, E. Moltchanova, G. Kindermann, and P. Scholtz. 2008. "GEO-BENE global database for bio-physical modeling v. 1.0 - concepts, methodologies and data. The GEO-BENE database report." In, 58. International Institute for Applied Systems Analysis (IIASA), Austria.

Valin, H., P. Havlík, A. Mosnier, M. Herrero, E. Schmid, and M. Obersteiner. 2013. 'Agricultural productivity and greenhouse gas emissions: trade-offs or synergies between mitigation and food security?', *Environmental Research Letters*, 8: 035019.

Valin, Hugo, Ronald D. Sands, Dominique van der Mensbrugghe, Gerald C. Nelson, Helal Ahammad, Elodie Blanc, Benjamin Bodirsky, Shinichiro Fujimori, Tomoko Hasegawa, Petr Havlik, Edwina Heyhoe, Page Kyle, Daniel Mason-D'Croz, Sergey Paltsev, Susanne Rolinski, Andrzej Tabeau, Hans van Meijl, Martin von Lampe, and Dirk Willenbockel. 2014. 'The future of food demand: understanding differences in global economic models', *Agricultural Economics*, 45: 51-67.

Williams, J.R. 1995. 'The EPIC Model.' in V.P. Singh (ed.), *Computer Models of Watershed Hydrology* (Water Resources Publications, Highlands Ranch, Colorado).
