# Supplementary material for: Enhanced agricultural carbon sinks provide benefits for farmers and the climate
Source: Nat Food. 2024 Sep 23;5(9):742–53. doi: 10.1038/s43016-024-01039-1 (PMC11420069; doi:10.1038/s43016-024-01039-1)
Supplement: Supplementary file 2 — Reporting Summary [file 43016_2024_1039_MOESM2_ESM.pdf]

Corresponding author(s): Stefan Frank
Last updated by author(s): Jul 11, 2024

Reporting Summary

Nature Portfolio wishes to improve the reproducibility of the work that we publish. This form provides structure for consistency and transparency in reporting. For further information on Nature Portfolio policies, see our Editorial Policies and the Editorial Policy Checklist.

Statistics

For all statistical analyses, confirm that the following items are present in the figure legend, table legend, main text, or Methods section.

n/a	Confirmed	
☒	☐	The exact sample size (*n*) for each experimental group/condition, given as a discrete number and unit of measurement
☒	☐	A statement on whether measurements were taken from distinct samples or whether the same sample was measured repeatedly
☒	☐	The statistical test(s) used AND whether they are one- or two-sided<br>*Only common tests should be described solely by name; describe more complex techniques in the Methods section.*
☒	☐	A description of all covariates tested
☒	☐	A description of any assumptions or corrections, such as tests of normality and adjustment for multiple comparisons
☒	☐	A full description of the statistical parameters including central tendency (e.g. means) or other basic estimates (e.g. regression coefficient) AND variation (e.g. standard deviation) or associated estimates of uncertainty (e.g. confidence intervals)
☒	☐	For null hypothesis testing, the test statistic (e.g. *F*, *t*, *r*) with confidence intervals, effect sizes, degrees of freedom and *P* value noted<br>*Give P values as exact values whenever suitable.*
☒	☐	For Bayesian analysis, information on the choice of priors and Markov chain Monte Carlo settings
☒	☐	For hierarchical and complex designs, identification of the appropriate level for tests and full reporting of outcomes
☒	☐	Estimates of effect sizes (e.g. Cohen's *d*, Pearson's *r*), indicating how they were calculated

Our web collection on statistics for biologists contains articles on many of the points above.

Software and code

Policy information about availability of computer code

Data collection	New agricultural CO2 sequestrations were parameterized in GLOBIOM as described in the method section by combining mainly datasets from Roe et al. (2021) and 3-PGmix simulations (version 0.1.6, https://github.com/trotsiuk/r3PG) and included in GLOBIOM for SSP2 (version 2.0, Fricko et al., 2018). A GitHub repository at https://iiasa.github.io/GLOBIOM/introduction.html provides the Global Biosphere Management Model (GLOBIOM) documentation, links to GLOBIOM resources, GAMS script descriptions, and dependency links that match the Trunk version of the GLOBIOM. Additional code can be made available upon request from the corresponding authors.
Data analysis	No software was used to analyze the data.

For manuscripts utilizing custom algorithms or software that are central to the research but not yet described in published literature, software must be made available to editors and reviewers. We strongly encourage code deposition in a community repository (e.g. GitHub). See the Nature Portfolio guidelines for submitting code & software for further information.

Data

Policy information about availability of data

All manuscripts must include a data availability statement. This statement should provide the following information, where applicable:

- Accession codes, unique identifiers, or web links for publicly available datasets
- A description of any restrictions on data availability
- For clinical datasets or third party data, please ensure that the statement adheres to our policy

The results that support the findings of the study are provided in the manuscript and the supplementary information. Source data are provided with this paper.

Human research participants

Policy information about studies involving human research participants and Sex and Gender in Research.

Reporting on sex and gender	N/A
Population characteristics	N/A
Recruitment	N/A
Ethics oversight	N/A

Note that full information on the approval of the study protocol must also be provided in the manuscript.

Field-specific reporting

Please select the one below that is the best fit for your research. If you are not sure, read the appropriate sections before making your selection.

☐ Life sciences ☐ Behavioural & social sciences ☒ Ecological, evolutionary & environmental sciences

For a reference copy of the document with all sections, see nature.com/documents/nr-reporting-summary-flat.pdf

Ecological, evolutionary & environmental sciences study design

All studies must disclose on these points even when the disclosure is negative.

Study description	We enhanced an economic spatially-explicit land use model with representation of agricultural CO2 sequestration options for assessment of cost-effective GHG abatement potentials and economic implications for farmers and ambitious climate stabilization scenarios.
Research sample	The key focus of the study is the land-use sector.
Sampling strategy	Based on a literature review we decided to include SOC enhancement, biochar application, and silvo-pastures as key agricultural CO2 sequestration technologies into GLOBIOM.
Data collection	Data was collected from existing literature and complemented with bio-physical modelling (3pgmix) and implemented in GLOBIOM for the economic assessment.
Timing and spatial scale	Model simulations were performed up to 2070. Markets, demand, and international trade are represented in the model at the level of 37 global regions. The supply side of the the model was run a 2 degree resolution.
Data exclusions	No data was excluded.
Reproducibility	We conducted a sensitivity analysis and compared our results to existing literature (derived from bottom-up studies).
Randomization	This is not relevant as the study conduced with a land-use model that represents the main land-use sector via production technologies but not individual actors/farmers.
Blinding	The parameterization and implementation of agricultural CO2 sequestration options in the model was performed by a subgroup of co-authors which also performed a first analysis of the results. All authors contributed then to further refining the approach and writing of the paper.

Did the study involve field work? ☐ Yes ☒ No

Reporting for specific materials, systems and methods

We require information from authors about some types of materials, experimental systems and methods used in many studies. Here, indicate whether each material, system or method listed is relevant to your study. If you are not sure if a list item applies to your research, read the appropriate section before selecting a response.

Materials & experimental systems

n/a	Involved in the study
☒	☐ Antibodies
☒	☐ Eukaryotic cell lines
☒	☐ Palaeontology and archaeology
☒	☐ Animals and other organisms
☒	☐ Clinical data
☒	☐ Dual use research of concern

Methods

n/a	Involved in the study
☒	☐ ChIP-seq
☒	☐ Flow cytometry
☒	☐ MRI-based neuroimaging
